# Investigation of a cluster of severe acute respiratory syndrome coronavirus 2 (SARS-CoV-2) infections in a hospital administration building

**DOI:** 10.1017/ice.2022.45

**Published:** 2023-02

**Authors:** Lucas D. Jones, Ernest R. Chan, Jennifer L. Cadnum, Sarah N. Redmond, Maria E. Navas, Trina F. Zabarsky, Elizabeth C. Eckstein, Jeffrey D. Kovach, Marlin Linger, Peter A. Zimmerman, Curtis J. Donskey

**Affiliations:** 1 Department of Molecular Biology and Microbiology, Case Western Reserve University School of Medicine, Cleveland, Ohio; 2 Cleveland Institute for Computational Biology, Case Western Reserve University, Cleveland, Ohio; 3 Research Service, Louis Stokes Cleveland VA Medical Center, Cleveland, Ohio; 4 Department of Medicine, University Hospitals Cleveland Medical Center, Cleveland, Ohio; 5 Pathology and Laboratory Medicine Service, Louis Stokes Cleveland VA Medical Center, Cleveland, Ohio; 6 Infection Control Department, Louis Stokes Cleveland VA Medical Center, Cleveland, Ohio; 7 Department of Population and Quantitative Health Sciences, Case Western Reserve University School of Medicine, Cleveland, Ohio; 8 The Center for Global Health & Diseases, Case Western Reserve University, Cleveland, Ohio; 9 Geriatric Research, Education, and Clinical Center, Louis Stokes Cleveland VA Medical Center, Cleveland, Ohio; 10 Case Western Reserve University School of Medicine, Cleveland, Ohio

## Abstract

**Objective:**

To investigate a cluster of severe acute respiratory syndrome coronavirus 2 (SARS-CoV-2) infections in employees working on 1 floor of a hospital administration building.

**Methods:**

Contact tracing was performed to identify potential exposures and all employees were tested for SARS-CoV-2. Whole-genome sequencing was performed to determine the relatedness of SARS-CoV-2 samples from infected personnel and from control cases in the healthcare system with coronavirus disease 2019 (COVID-19) during the same period. Carbon dioxide levels were measured during a workday to assess adequacy of ventilation; readings >800 parts per million (ppm) were considered an indication of suboptimal ventilation. To assess the potential for airborne transmission, DNA-barcoded aerosols were released, and real-time polymerase chain reaction was used to quantify particles recovered from air samples in multiple locations.

**Results:**

Between December 22, 2020, and January 8, 2021, 17 coworkers tested positive for SARS-CoV-2, including 13 symptomatic and 4 asymptomatic individuals. Of the 5 cluster SARS-CoV-2 samples sequenced, 3 were genetically related, but these employees denied higher-risk contacts with one another. None of the sequences from the cluster were genetically related to the 17 control sequences of SARS-CoV-2. Carbon dioxide levels increased during a workday but never exceeded 800 ppm. DNA-barcoded aerosol particles were dispersed from the sites of release to locations throughout the floor; 20% of air samples had >1 log_10_ particles.

**Conclusions:**

In a hospital administration building outbreak, sequencing of SARS-CoV-2 confirmed transmission among coworkers. Transmission occurred despite the absence of higher-risk exposures and in a setting with adequate ventilation based on monitoring of carbon dioxide levels.

Healthcare personnel are at risk to acquire severe acute respiratory syndrome coronavirus 2 (SARS-CoV-2) from infected patients or coworkers.^1–5^ In hospitals, infection control measures including universal masking, use of personal protective equipment during patient care, and preadmission screening are commonly implemented to minimize the risk for acquisition of SARS-CoV-2.^3–5^ Hospitals also have ventilation requirements that may substantially lower the risk for airborne transmission compared to many commercial and residential buildings.^6,7^ These measures reduce, but do not eliminate, the risk for SARS-CoV-2 transmission. For example, transmission has been reported when patients with coronavirus disease 2019 (COVID-19) are not recognized at the time of admission.^2,5^ Transmission of SARS-CoV-2 from infected personnel to coworkers may also occur despite universal masking,^2,8^ including in ancillary care areas and outpatient clinics.^9^

Healthcare personnel with no direct patient care duties often work in office buildings separate from the main hospital. As noted previously, ventilation requirements in these buildings are less stringent than in hospitals.^6,7^ In addition, working in cubicles might present an increased risk of airborne transmission in comparison to working in offices with doors closed. In South Korea, an outbreak of COVID-19 cases was reported in a call center on 1 floor of a commercial–residential building.^10^ In the current study, we investigated a cluster of 17 COVID-19 cases that occurred among coworkers on 1 floor of a hospital administration building. Whole-genome sequencing was performed to determine the relatedness of the viral specimens. We monitored carbon dioxide levels to assess ventilation, and we examined the potential for airborne dispersal of aerosols by assessing the dispersal of DNA-barcoded aerosol test particles.

## Methods

### Study setting

The Louis Stokes Cleveland VA Medical Center is a 215-bed acute-care hospital with an affiliated 250-bed long-term care facility. The outbreak occurred on the fourth floor of an administration building adjacent to the hospital; no similar clusters were detected on the other floors of the building. The fourth floor of the building has 18 offices, ∼90 in-use cubicles, central bathrooms for men and women, and a shared break room. The total area of the floor is 392.4 m^2^ (4,224 square feet). During the outbreak period, ∼100 total personnel worked on the floor on weekdays. The workspaces were arranged to provide at least 2 m distance between coworkers. The ventilation system for the building was designed to provide 6 air changes per hour. In response to the COVID-19 pandemic, the ventilation system was modified to increase the percentage of outdoor air to 25% or higher if feasible depending on weather conditions.^6,7^ Fresh and recirculated air passed through filters with a minimum efficiency reporting value (MERV) of 13 or greater.

Personnel were required to wear medical face masks unless in a workspace behind closed doors. Recommendations were made for personnel to eat meals alone at their desks and to maintain physical distancing. Eye protection with goggles or face shield was recommended in situations where it was not possible to maintain physical distancing for 15 minutes or more. All personnel were screened for COVID-19 symptoms and fever on entry to the facility. Testing for SARS-CoV-2 was recommended for personnel with symptoms of COVID-19, including mild symptoms such as sore throat and nasal congestion.

### Contact tracing investigation

The study protocol was approved by the Cleveland VA Medical Center’s Institutional Review Board. The infection control department initiated an investigation on December 28, 2020, after 4 COVID-19 cases were diagnosed in employees working on the floor. Contact tracing was conducted in accordance with Centers for Disease Control and Prevention (CDC) recommendations.^1^ Higher-risk exposures were defined as 15 minutes or more of continuous or cumulative contact over 24 hours within 1.8 m without wearing both a face mask and eye protection.^1^ Personnel were questioned regarding contacts with coworkers both at work and in the community. Surveillance nasopharyngeal swab testing was recommended for all personnel on the floor beginning on January 2, 2021, and it was completed on January 20, 2021.

### SARS-CoV-2 sequencing and data analysis

RNA was extracted from positive nasopharyngeal swab specimens with the QIAamp Viral RNA Mini Kit (Qiagen, Hilden, Germany) according to the manufacturer’s instructions. For comparison, we sequenced 17 samples from hospital employees or hospitalized patients with COVID-19 between November 2020 and January 2021. Sequences were aligned to the reference COVID-19 sequence (NCBI accession: NC_045512.2) using Bowtie2 software. Variant positions were defined as any genomic position that had an alternate allele frequency >50% in any of the sequenced samples and were determined using samtools and mpileup software tools.^11,12^ Sequences were assigned to a transmission cluster if they had <2 base differences based on their variant positions.^4^ The consensus sequences for each strain were classified into lineages using Pangolin software (github.com/cov-lineages/pangolin).

### Assessment of ventilation based on monitoring of carbon dioxide levels

Based on recommendations from the Centers for Disease Control and Prevention (CDC), carbon dioxide readings >800 ppm are considered an indicator of suboptimal ventilation in buildings requiring intervention.^13^ To assess the adequacy of ventilation on the floor of the building where the cluster occurred, we measured carbon dioxide levels during a typical workday. In one open workspace with cubicles and in the woman’s bathroom, carbon dioxide levels were continuously monitored from 4 a.m. to 8 p.m. using an IAQ-MAX CO_2_ Monitor and Data Logger (CO2 Meter, Ormond Beach, FL) with readings logged every 1 minute. In addition, carbon dioxide levels were measured in multiple locations on the floor during the afternoon to determine whether levels varied by location.

### Dispersal of DNA-barcoded aerosol particles

DNA-barcoded aerosol test particles provide a means to assess dispersal and clearance of aerosols in buildings and other enclosed spaces.^14^ We released veriDART DNA-barcoded aerosol test particles (SafeTraces, Pleasanton, CA) in 5 locations on the floor; each of the aerosols had a unique DNA barcode. A hand-held Bucko sprayer (SafeTraces) was used to release 11 mL of droplets containing the aerosol particles over 1 minute; based on particle counter readings, the device disperses predominantly 0.5- to 10-µm liquid droplets.^14^ Air samples were collected from 10 locations on the floor for 30 minutes after release with an Airlite air sample pump (SKC, Eighty Four, PA). During the aerosol release and air sampling period, the doors of all offices and the breakroom were open, but the bathroom door was closed. For the purposes of the study, detection of particles in air collected in locations outside the immediate release area was considered an indication of airborne dispersal; detection of >1 log_10_ aerosol particles was considered an indication of dispersal with suboptimal clearance of particles over the 30-minute collection period.

## Results

### Contact tracing investigation

**Fig. 1.**
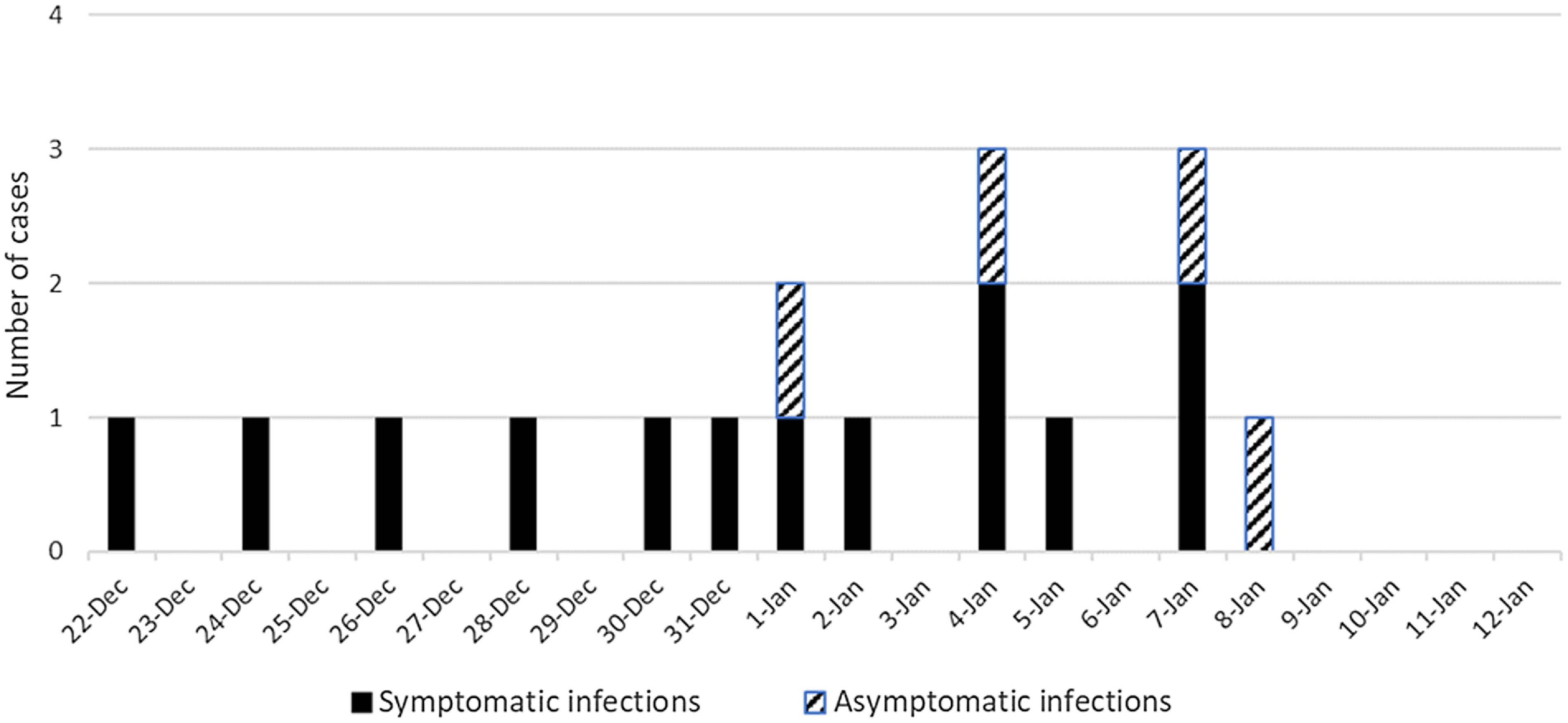
Epidemic curve for the cluster of COVID-19 cases on 1 floor of a hospital administration building. Day 1 is December 22, 2020, and day 18 is January 8, 2021.

Figure 1 shows the epidemic curve for the cluster of COVID-19 cases. During an 18-day period from December 22, 2020, through January 8, 2021, 17 employees had positive RT-PCR results for SARS-CoV-2 from nasopharyngeal samples, including 13 with symptomatic COVID-19 infections. Of 83 asymptomatic individuals recommended for surveillance screening, 80 (96%) were screened by nasopharyngeal swab RT-PCR and 4 (5%) tested positive. The only common areas used by all employees were the bathrooms and the breakroom. None of the employees reported 15 minutes of continuous close contact with an infected coworker in work areas or in the breakroom. Three symptomatic employees reported higher-risk community exposures within 1 week prior to their COVID-19 diagnoses, including cases identified on December 22, 2020, December 26, 2020, and January 1, 2021.

### Sequencing analysis

**Fig. 2.**
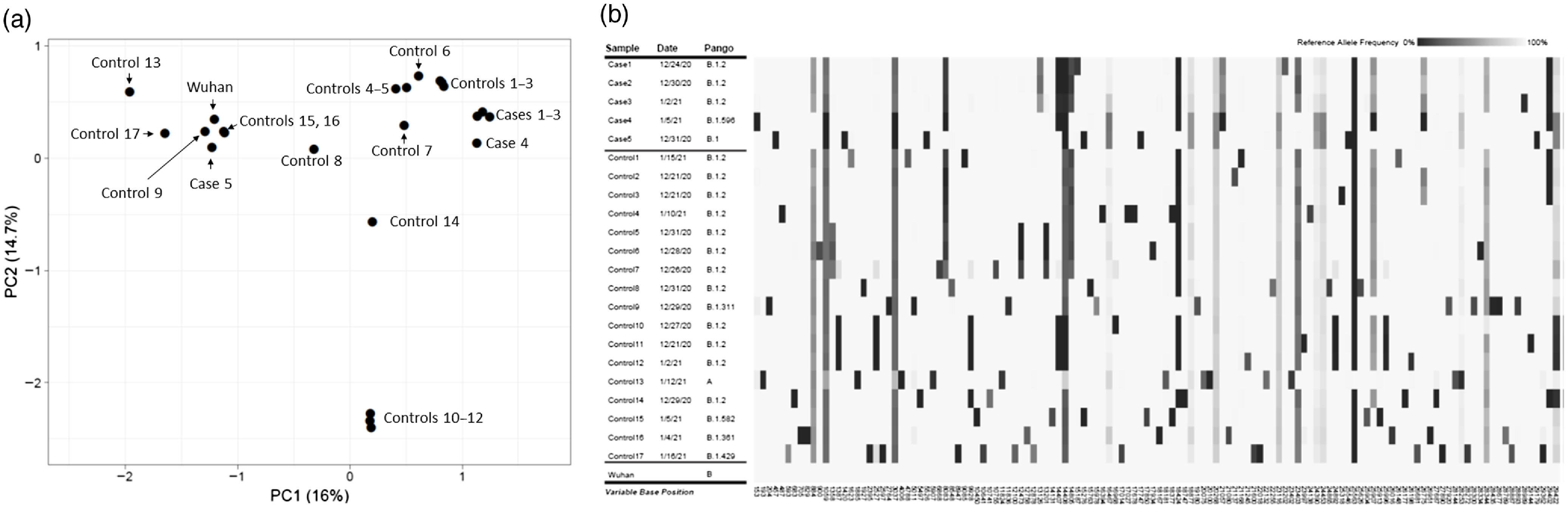
Principal component analysis (A) and genetic variant profiles (B) of the sequenced SARS-CoV-2 relative to the date of sample collection and the Wuhan-Hu-1 reference genome. For comparison, 17 additional sequences from patients or employees diagnosed with COVID-19 at the Cleveland VA Medical Center between December 21, 2020, and January 16, 2021, are shown. Gradient intensity of the bars in the genetic variant profiles indicates the reference allele frequency at that position.

Of the 17 employees with COVID-19, 10 had samples available for sequencing, including 4 from asymptomatic individuals and 6 with mild COVID-19. Of the 10 samples sequenced, 5 had minimum coverage of >95% of the genome at a depth of at least 78× coverage depth and were included in the analysis: 4 with mild COVID-19 and 1 asymptomatic individual. The average cycle threshold for these 5 samples was 31.2 (range, 27.8–37.8). The average cycle threshold for the 5 samples that did not have adequate coverage was 38.8 (range, 33.2–42.4). Figure 2 shows the principal component analysis and genetic variant profiles of the sequenced SARS-CoV-2 relative to the date of sample collection and the Wuhan-Hu-1 reference genome; for comparison, 17 additional sequences from patients or employees diagnosed with COVID-19 at the Cleveland VA Medical Center between December 21, 2020, and January 16, 2021, are shown.

Principal components analysis of the genetic profile of the cohort revealed that 3 of the employees (cases 1–3, 2 females and 1 male) clustered tightly suggesting that they were infected with genetically related SARS-CoV-2 from the Pangolin B.1.2 phylogenetic lineage with 1 to 3 pairwise nucleotide differences. The supplemental material (online) provides further information on lineage naming and on the diversity of the B.1.2 lineage, including shared mutations defining the B.1.2 lineage and numerous additional reported polymorphisms. The other 2 employees (cases 4 and 5) had genetically distinct SARS-CoV-2 from the Pangolin B.1 and B.1.596 lineages. Cases 4 and 5 differed from cases 1–3 by 15 and 6–21 pairwise differences, respectively.

**Fig. 3.**
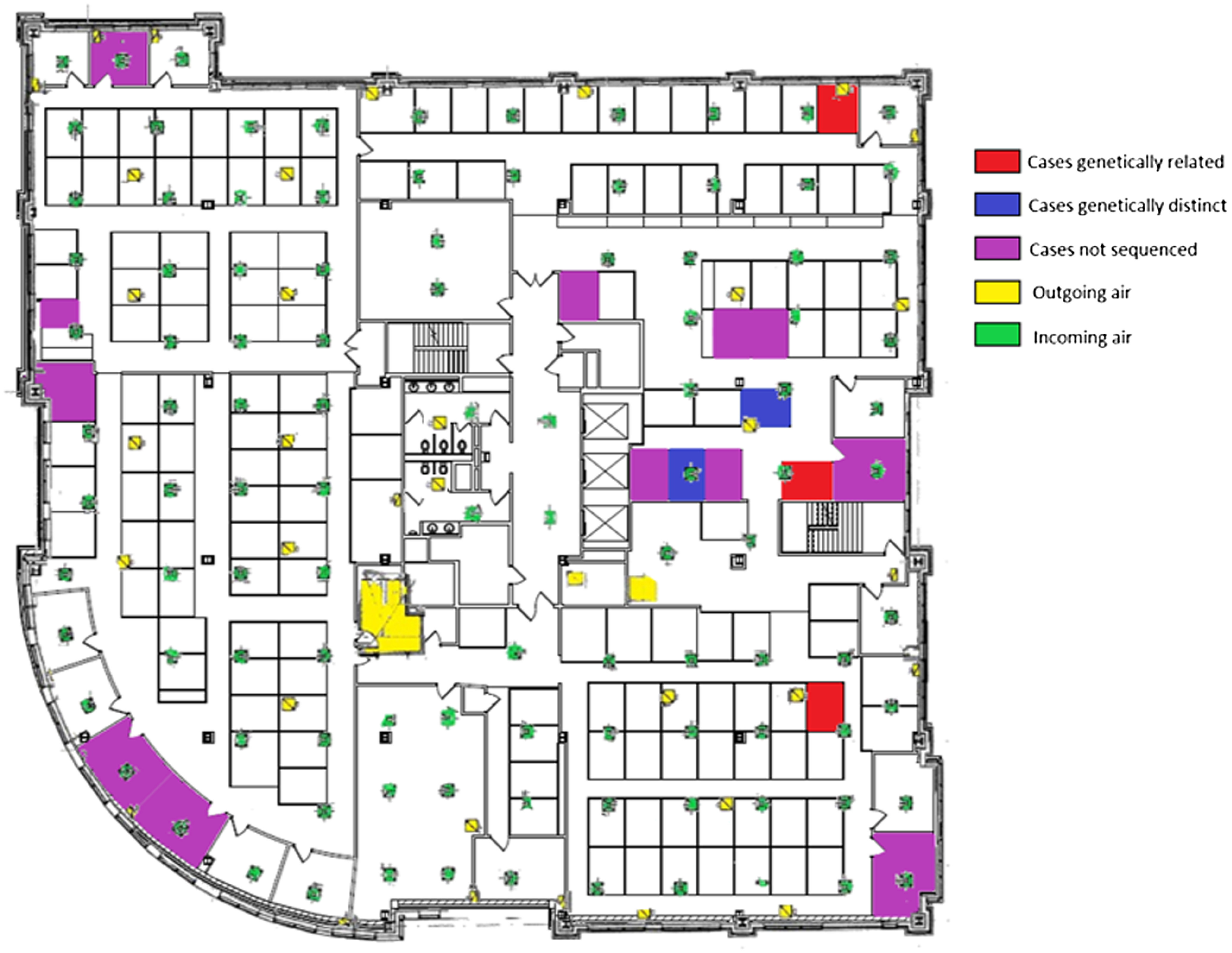
Work locations the 17 employees with COVID-19 including the 5 individuals whose viruses were successfully sequenced. The 3 individuals with genetically related SARS-CoV-2 were diagnosed on December 24, 2020, December 30, 2020, and January 1, 2021. Yellow indicates sites of outgoing air ventilation ducts and green indicates incoming air ventilation ducts.

Figure 3 shows the work locations all 17 infected employees, including the 5 individuals whose viruses were sequenced. The 3 employees with genetically related SARS-CoV-2 worked in separate departments and did not report any interactions with one another prior to their COVID-19 diagnoses on December 24, 2020, December 30, 2020, and January 2, 2021, respectively. The 17 control SARS-CoV-2 samples analyzed for comparison with the employees had SARS-CoV-2 sequences that were distinct from the cluster sequences.

### Carbon dioxide monitoring

During a typical workday on the floor, the carbon dioxide level in areas with open cubicles was ∼400 ppm prior to arrival of personnel at 7 a.m., then it rose to a peak level of 769 ppm at 2:45 p.m., with a subsequent decrease as the number of personnel decreased beginning at 3:30 p.m. The number of personnel present on the floor was ∼100 during the middle of the workday. Similar carbon dioxide levels were obtained from a carbon dioxide monitor placed inside a bathroom from 4 a.m. to 8 p.m., and individual readings obtained from multiple locations on the floor were equivalent during a workday (data not shown).

### Dispersal of DNA-barcoded aerosol particles

**Fig. 4.**
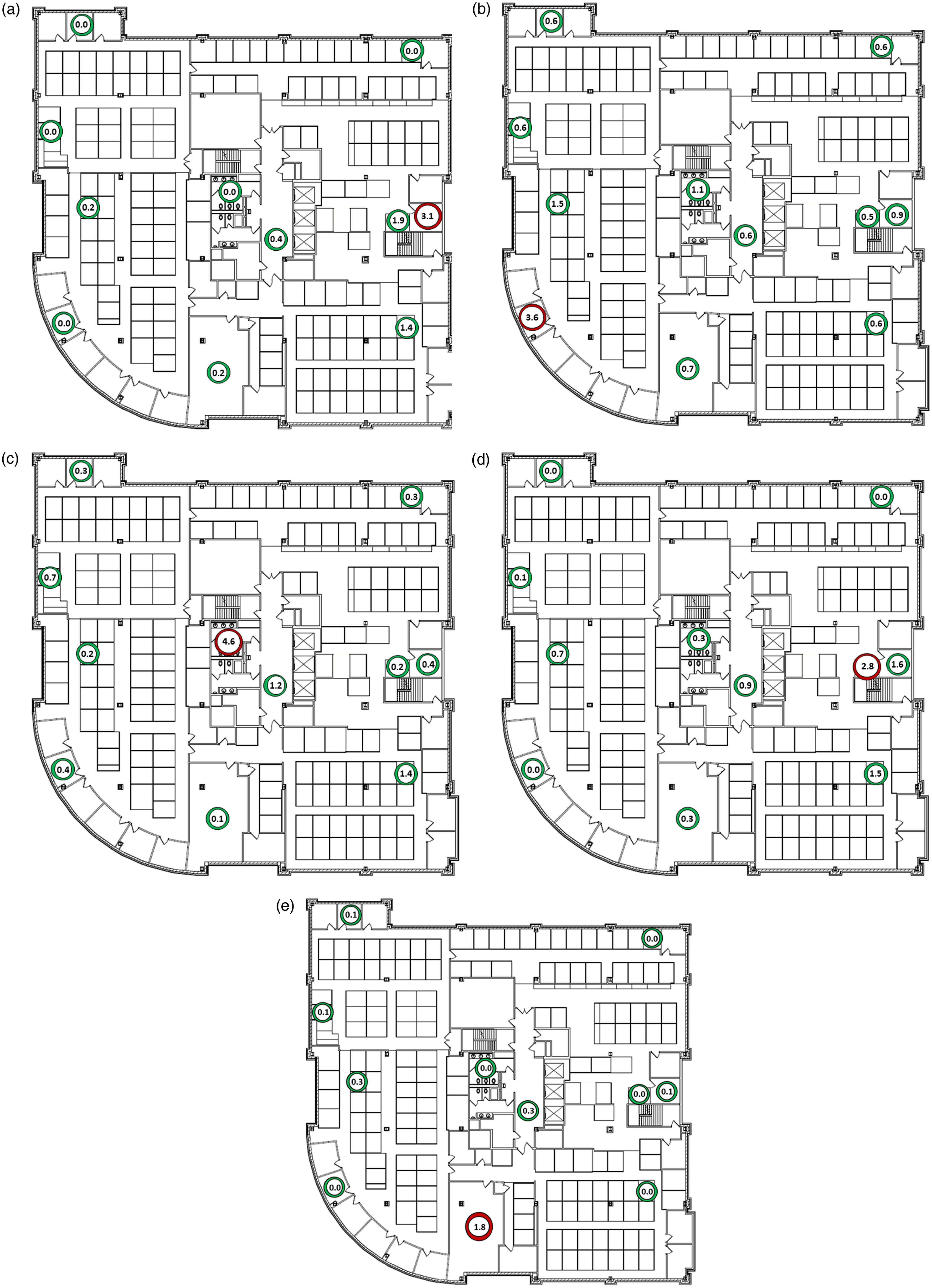
Log_10_ DNA-barcoded particles detected in air samples collected from multiple sites during a 30-minute period after release of the particles from 2 offices (A and B), a restroom (C), a cubicle (D), and a breakroom (E). The red circle shows the site of particle release and the green circles show the sites of air collection.

Figure 4 shows the log_10_ particles detected in air samples collected from multiple sites during a 30-minute period after release of DNA-barcoded particles from 2 offices (A and B), a bathroom (C), a cubicle (D), and the breakroom (E). Between 1.8 and 4.6 log_10_ particles were detected at the release sites; the highest concentration was detected in the bathroom, which was the only release site with the door closed. The released particles were detected in between 5 (50%) of 10 and 10 (100%) of 10 air-sampling sites outside the release site. However, for each of the particles released from the offices, bathroom, and cubicle, only 2 (20%) of the 10 air sampling sites had >1 log_10_ aerosol particles detected, and most were in proximity to the release site. For the particles released from the breakroom, none of the air sampling sites had >1 log_10_ aerosol particles detected.

## Discussion

In the current investigation, sequencing analysis provided support for transmission of SARS-CoV-2 among 3 coworkers on 1 floor of a hospital administration building. The SARS-CoV-2 sequences of these 3 individuals were distinct from the sequences of 2 coworkers who potentially had community acquired viral variants and 17 control sequences from patients and employees with COVID-19 during the same period. The sequences from the 3 administrative employees, as well as many of the control sequences, belonged to the B.1.2 lineage, which was transmitted widely in the United States during the fourth quarter of 2020. Transmission occurred despite universal masking and physical distancing of at least 2 m for all workspaces. Moreover, the 3 employees with genetically related SARS-CoV-2 worked in separate departments and did not report any interactions with one another prior to their COVID-19 diagnoses. These findings highlight the substantial risk for transmission of SARS-CoV-2 among coworkers in office buildings despite standard control measures and suggest possible airborne transmission.

The CDC recommends that building managers take efforts to ensure adequate ventilation to reduce the risk for SARS-CoV-2 transmission.^6,7^ The administration building ventilation system provided 6 air changes per hour with MERV 13 or greater filtering of recirculated air. In response to the pandemic, the percentage of outdoor air was increased.^6,7^ Carbon dioxide monitoring demonstrated that the ventilation provided was sufficient to maintain levels below 800 ppm during a typical workday, suggesting adequate ventilation based on current CDC recommendations.^13^ In several recent reports, hospitalized patients with COVID-19 not detected on admission have transmitted SARS-CoV-2 to roommates despite ventilation requirements of 6 or more total air changes per hour and often in the absence of exposure to aerosol-generating procedures.^5,15,16^ These reports and the cluster reported here raise concern that airborne transmission might occur when individuals share the same enclosed space for prolonged periods despite ventilation that meets current standards.

The widespread dispersal of DNA-barcoded aerosols demonstrates the potential for airborne dispersal of viral particles on the floor of the building where the cluster occurred. Such dispersal could have occurred through direct air movement among workspaces or through recirculated air that was not adequately filtered. Further studies are needed to determine the correlation between dispersal of low concentrations of DNA-barcoded aerosol particles and the potential for dispersal of respiratory aerosols containing viable virus particles.

Our study had several limitations. We did not sequence all viruses from the cluster because some samples were not available or did not meet the requirements for quality of sequencing. Thus, we cannot exclude the possibility that the presumed transmission events did not represent concurrent acquisition of related viruses circulating in the community or transmission from asymptomatic or symptomatic coworkers whose samples were not sequenced. However, the fact that the sequences of 17 control COVID-19 cases were distinct from the 3 coworkers with genetically related SARS-CoV-2 provides support for transmission. We considered carbon dioxide levels below 800 ppm to be an indicator of adequate ventilation based on CDC recommendations. However, others have recommended that carbon dioxide levels in buildings should not exceed 700 ppm to minimize risk for airborne transmission of SARS-CoV-2.^17,18^ We did not assess the impact of interventions such as use of portable high efficiency particulate air (HEPA) air cleaners on dispersal of the DNA-barcoded aerosol particles. Finally, we did not have data on compliance of personnel with facemasks and social distancing.

In conclusion, our investigation provides evidence of possible transmission of SARS-CoV-2 among coworkers in a hospital administration building. The purported transmission occurred despite universal masking and physical distancing in a setting with evidence of adequate ventilation. Additional studies are needed to evaluate the risk for airborne dissemination in office buildings and to identify effective control measures.
